# The Relationship Between Peri-Implant Marginal Bone Loss and Resonance Frequency Analysis

**DOI:** 10.3390/jfb16020071

**Published:** 2025-02-18

**Authors:** Esteban Pérez-Pevida, Iván Monteagudo-Villalobos, David Chávarri-Prado, Alejandro Estrada-Martínez, Miguel Beltrán-Guijarro, Markel Diéguez-Pereira, Aritza Brizuela-Velasco

**Affiliations:** 1 DENS-ia Research Group, Faculty of Health Sciences, Miguel de Cervantes European University, 47012 Valladolid, Spain; aritzabrizuela@hotmail.com; 2 Department of Surgery, Faculty of Medicine, University of Salamanca, 37007 Salamanca, Spain; 3 Faculty of Health Sciences, Miguel de Cervantes European University, 47012 Valladolid, Spain; monteagudo.dds@gmail.com (I.M.-V.); davidchavarri@hotmail.com (D.C.-P.); alex.estra12@gmail.com (A.E.-M.); markeldieguez@hotmail.com (M.D.-P.); 4 Department of Surgery, Faculty of Sports and Health Sciences, University of Zaragoza, 22006 Huesca, Spain; mbeltgui@gmail.com

**Keywords:** dental implants, bone resorption, peri-implantitis, resonance frequency analysis

## Abstract

Resonance frequency analysis (RFA) has been used as a diagnostic method to measure implant stability at all stages of healing. In addition to evaluating the status of the peri-implant marginal bone, it can also indicate the most appropriate time to load the implant. This in vitro study aimed to evaluate the efficacy of RFA as a diagnostic method for the detection of peri-implant marginal bone loss (MBL). Forty bone-level Klockner Vega implants were placed in a polyurethane block with elastic properties similar to those of the maxillary bone. The insertion torque and primary implant stability at the time of placement were measured using an RFA device. A circumferential peri-implant defect was created by removing the cortical bone portion in each implant using a trephine. The stability values were measured again using RFA. The stability values measured using RFA were lower after the creation of the circumferential peri-implant defect, indicating a statistically significant decrease in implant stability. The results of the study tend to show a relationship between peri-implant marginal bone loss and modifications in implant stability measured by RFA.

## 1. Introduction

Dental implant treatment has exhibited high long-term predictability, with 5- and 10-year survival rates of 97% [1] and 94% [2], respectively. Notably, studies with a follow-up period of up to 20 years have reported survival rates of around 90% [3,4]. However, survival of the implant does not indicate treatment success. Dental implant treatment may be complicated by mechanical–technical, biological, and aesthetic complications. Such complications have been reported in close to 8% of cases [2]; however, this rate varies depending on the criteria used for evaluation and the follow-up period [5]. The Albretksson criteria have been used to determine the success of implant treatment in most cases [6].

Peri-implant disease, the primary cause of biological failure after post-loading, can be classified as mucositis and peri-implantitis [7,8]. Peri-implantitis is defined as “a pathological situation associated with bacterial plaque produced in the tissues surrounding dental implants, characterized by inflammation of the peri-implant mucosa (PIM) with subsequent progressive loss of the supporting bone” [7,8]. Mucositis differs from peri-implantitis in that no bone loss is observed in patients with mucositis. The definition of peri-implantitis was defined and accepted in the last Workshop of the European Federation of Periodontology (EFP) and the American Association of Periodontology (AAP).

Peri-implant disease is, without a doubt, the most frequently encountered complication of implant treatment in daily practice. The choice of diagnostic method used for the detection of peri-implantitis remains controversial. Light probing remains the most widely used method for the evaluation of bleeding and suppuration, whereas radiographic examination is the choice of diagnostic method for the detection of bone loss [9,10].

In the most recent systematic reviews and meta-analysis, a mean prevalence of peri-implantitis of 18% at the patient level and 12% at the implant level were reported, similar to those found in other studies (17%–11%, respectively) [11,12,13,14]. However, the literature is disparate in this regard; other studies report higher peri-implantitis prevalences of around 30–40% [15]. A cross-sectional study carried out in collaboration with the Spanish Society of Periodontology highlighted the prevalence of peri-implantitis in 474 implants placed nationwide, finding rates of 14% and 11% at patient and implant level, respectively, according to the current definition of peri-implantitis [16]. Some authors consider bleeding on probing as a representative clinical pattern of peri-implantitis, when in fact it is an index with a high false positive ratio [17,18]. Others also consider probing depth to be representative of the development of peri-implantitis, alone or in combination with bleeding on probing. Probing depth in implants is greater than in teeth, as there is a different histological composition and, therefore, different resistance to probe penetration [19]. However, it has been shown that, although probing depth is not related to the development of peri-implantitis, it is an indicator of the level of bone crest in implants with peri-implantitis, in which cases the probe tip may exceed a mean of 0.5 mm beyond the connective tissue level [20].

The fact that a large number of healthy implants also show bleeding on probing may be due, in part, to the fact that probing implants may cause non-specific bleeding that is not necessarily related to the possible inflammatory state of the peri-implant tissues [21]. Furthermore, the evidence concludes that the peri-implant mucosa has a higher pro-inflammatory state than the periodontal mucosa [22].

Although periapical radiography with the use of a positioner is the recommended method for the assessment of marginal bone loss [23], there are studies that demonstrate its possible biases when assessing not only the amount of interproximal bone loss—approximately underestimated by 1 mm of marginal bone loss [24,25]—but also the type of peri-implant defect [23]. In addition, due to its minimal but existing radiation potential, we can consider its repetitive use as an invasive method of diagnosing peri-implant pathology.

Several invasive and non-invasive methods have been proposed for determining implant stability owing to this controversy [11]. Resonance frequency analysis (RFA) is the most widely used non-invasive method for evaluating the bone-implant bond [26,27,28]. It is a highly reproducible method that facilitates the monitoring of implant stability and marginal bone loss [29].

RFA, introduced by Meredith et al. in 1996 for the measurement of implant stability, responds to the physical phenomenon of resonance to establish the natural frequency of vibration of the implant by emitting a sinusoidal signal to a transducer screwed to the implant [30]. Over several generations of improvements, it was possible to adapt these physical principles to a clinical environment to make them easier to understand and handle. In third-generation devices, this resonance is translated into implant stability quotient (ISQ) values ranging from 0 to 100. The total effective length over the marginal bone and its loss affects the measurement of RFA. From a clinical point of view, RFA values between 50 and 70 are relevant, with values around the latter value indicating good primary stability.

Despite the limited evidence in the current literature on the use of RFA, some in vitro studies have highlighted a decrease in ISQ values in simulated peri-implant defects, especially in circumferential ones [29]. Schwarz and coworkers stated that although it is possible to describe different types of defects, the surgical entry at peri-implantitis sites often reveals a circumferential pattern of bone loss [31]. In addition, the possibilities of treatment by regeneration of this type of circumferential defect may be more favorable than for other types, as they are more self-contained and have more possibilities for vascularization compared to defects where one or more walls are missing. However, the prognosis is related to the size of the defect and thus to the possibility of early diagnosis. In this sense, it has been observed in some studies that the decrease in RFA was more pronounced in the first 2 mm of marginal bone loss [32], revealing a potential tool for the early diagnosis of initial peri-implantitis cases and the prevention of the onset of the disease.

Based on the results of previous studies, it was hypothesized that resonance frequency analysis could be used to determine any changes in the peri-implant contour during the monitoring of implant stability. Therefore, this trial study aimed to evaluate the efficacy of RFA as a method for detecting peri-implant marginal bone loss, determined by the variability of the ISQ results from the test.

## 2. Materials and Methods

### 2.1. Implant Design

Fifty Klockner Vega RV (Klockner SA, Barcelona, Spain) bone-level implants measuring 4 mm in diameter and 10 mm in length to the apex were used in this study. Klockner Vega is a parallel wall-shaped implant with a flat apex and a conical internal and platform-switched connection, with a surface treatment across the entire model.

### 2.2. Simulation of Native Bone and Implant Insertion Protocol

A polyurethane block (Sawbones, Pacific Research Laboratories Inc., Washington, DC, USA), with a density of 0.32 gr/cm^3^, and an outer layer of 1 mm thickness, with a density of 1.64 gr/cm^3^, was used in the study to replicate the elastic properties of the human maxillary bone, comprising cortical and cancellous bone and similar to a Type III or Type IV bone in the Lekholm and Zarb bone quality classification [32]. The manufacturer of the sawbones indicates that the resin of two densities used has a flexural modulus of 5.1 and 12 GPa, thus being close to that corresponding to the maxillary bone, which ranges from 2 to 15 GPa (trabecular and cortical, respectively).

The block was delimited in fifty identical regions, each one with an area of sixteen square millimeters in order to keep an optimal distance between implants.

**Figure 1:**
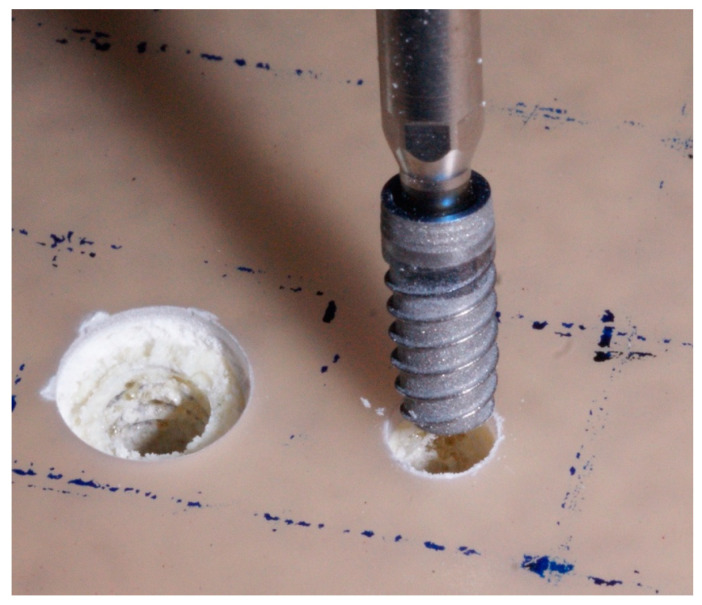
Dental implant placement.

The implants were placed at the juxta-osseous level, in accordance with the drilling protocol recommended by the manufacturer (Klockner SA, Barcelona, Spain) for implants with a diameter of 4.0 mm (Figure 1). In an attempt to simulate the realistic clinical situation, drilling was performed using the initial and pilot bur at 1200 rpm and the rest of the burs at 600 rpm with a saline solution using an electronic surgical unit (W&H, Dentalwerk, Austria), and the implants were inserted without a saline solution at 25 rpm.

### 2.3. Simulation of Peri-Implant Bone Defect

**Figure 2:**
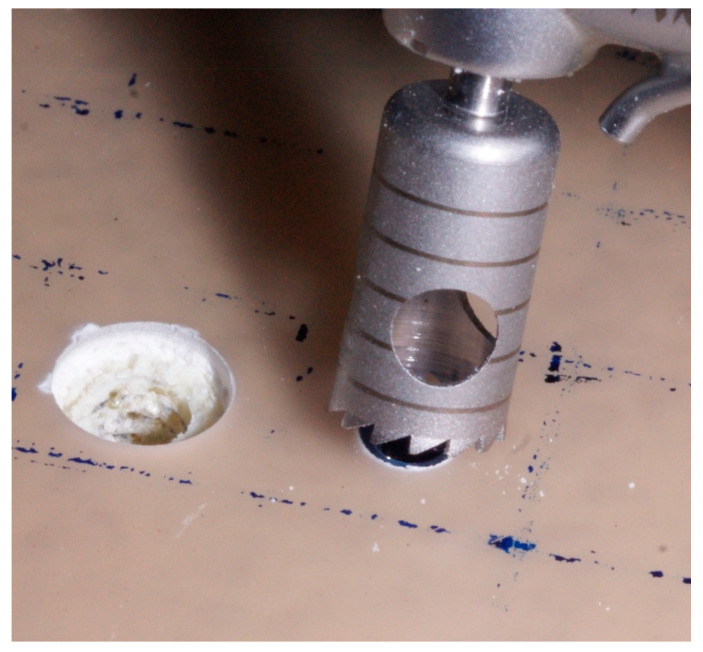
The trephine used for bone defect simulation.

**Figure 3:**
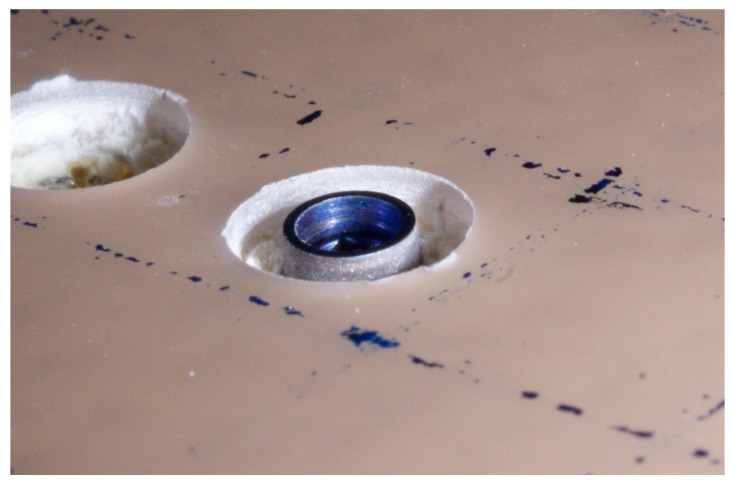
Peri-implant bone defect simulation.

A baseline measurement of primary stability was performed after simulating circumferential peri-implant bone defects with a depth of 2 mm and a diameter of 8 mm using a trephine (Meisinger, Neuss, Germany) (Figure 2). The horizontal and vertical sizes of the defects were the same in all cases (Figure 3). The creation of a defect with a homogeneous width was ensured by using the same trephine for all specimens, with an exact inner diameter of 8 mm, whose geometric center was aligned with that of the implant. The depth of 2 mm for each defect was ensured by referring to the first depth line of the trephine itself. Implant placement, defect creation, and measurements were performed by a single operator for standardization.

### 2.4. Measurement of Stability

Two noninvasive methods were used to measure the stability of the implant. The final insertion torque value of each implant was recorded using a 100 N digital torque calibrator (Mecmesin, Barcelona, Spain), and the primary stability of each implant was monitored using a third-generation Osstell ISQ RFA device (Osstell AB, Gothenburg, Sweden). A different titanium metal transducer (MultiPeg Driver, Integration Diagnostics AB, Gothenburg, Sweden) was orthoradially manually screwed to 10 Newtons (N) into place for each implant as an offset cantilever piece, and the stability was measured in two directions perpendicular to the longitudinal axis of the implant simulating the buccal–lingual (BL) direction and the mesiodistal (MD) direction.

**Figure 4:**
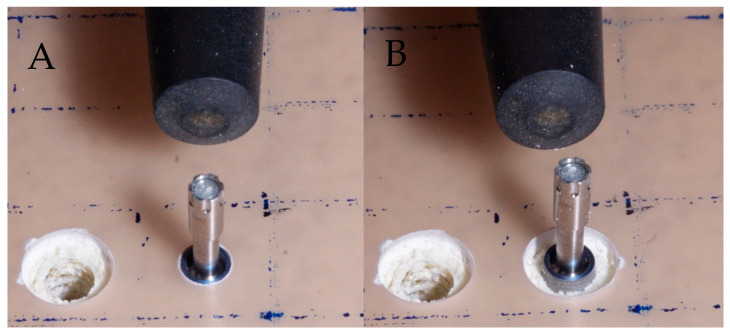
Measurement of primary implant stability before (**A**) and after (**B**) defect simulation, measured on the same implant.

The measurements were performed according to the manufacturer’s suggested technique, with the probe being held perpendicular to the MultiPeg until the recording warning sound was heard. The MultiPeg used for the 4.0 mm Klockner Vega implant was the MultiPeg 26. A trephine was used at 1200 rpm to create a circumferential peri-implant defect in each implant placed in the polyurethane block by removing a cortical bone portion 8 mm wide and 2 mm deep. The measurement of the stability values was repeated in the BL and MD directions (Figure 4). The implant placement and simulation of the peri-implant defects were carried out with a minimum defect spacing of 10 mm.

### 2.5. Statistical Analysis

The primary response variable was defined as the difference between the RFA values measured at the time of implant placement and those measured after the creation of peri-implant defects. The secondary response variable was defined as the relationship between the insertion torque and initial RFA values. The quantitative variables, i.e., the RFA and insertion torque values, are presented as the mean (measure of central tendency) and standard deviation (measure of dispersion). The Wilcoxon signed-rank test was used to assess the differences in the quantitative variables. Spearman’s correlation coefficient, with results ranging from −1 to 1, was used to evaluate the possible relationship between the insertion torque and initial RFA value. The level of significance was set at 5%. The null hypothesis (Ho) of no statistically significant differences being observed between the RFA values measured at the time of implant placement and those measured after the creation of peri-implant defects was rejected when the *p*-value in the corresponding average contrast test was <0.05. All the *p*-values are two-tailed. The 95% confidence intervals (CIs) were calculated using the bootstrap resampling method. All the statistical analyses were performed using SPSS version 21 software (SPSS, Chicago, IL, USA).

## 3. Results

A total of 50 bone-level parallel wall-shaped implants were placed in the same polyurethane block.

None of the 50 implants placed in the polyurethane block were rejected owing to a lack of stability (less than 35 newtons insertion torque). Thus, the primary stability values of all 50 implants that were placed initially were recorded.

As shown in Table 1, the mean implant stability quotient (ISQ) value at the time of implant placement was 72.2. The mean ISQ values in the buccal–lingual (BL) and mesiodistal (MD) directions were 72.23 ± 1.81 and 72.25 ± 2.20, respectively, indicating no statistically significant differences between the measurements. The insertion torque at the time of the implant placement was 32.38 ± 6.62 newtons.

**Figure 5:**
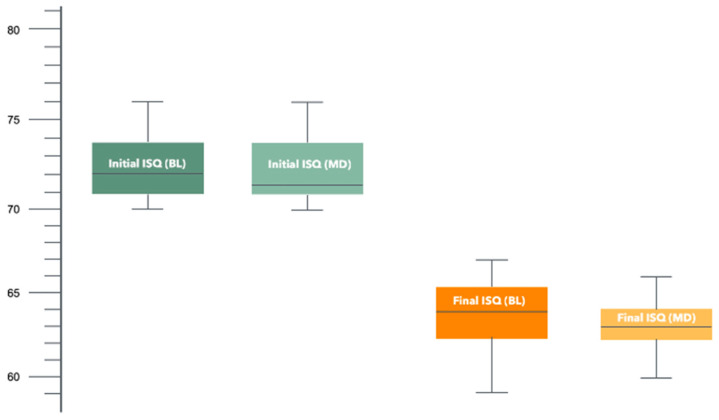
Boxplot diagram of the AFR stability values (ISQ), initial and final, in the two measurement directions.

The mean ISQ value after the induction of circumferential peri-implant defects was 63.3. The mean ISQ values in the BL and MD directions were 63.38 ± 1.76 and 63.38 ± 1.49, respectively, indicating no statistically significant differences between the measurements (Figure 5).

The difference between the ISQ values measured at the time of implant placement and those measured after the creation of the circumferential peri-implant defects was 8.8. This difference was 8.85 ± 1.87 and 8.87 ± 2.31 in the BL and MD directions, respectively. The difference between the baseline ISQ values and the values after the creation of the defects was statistically significant (*p* < 0.001) (Table 1).

A minimal, but statistically insignificant, positive correlation was observed between the insertion torque and initial RFA values at the implant placement (r ≠ 0; *p* > 0.05).

The insertion torque at the time of the implant placement was 32.38 ± 6.62 Ncm. A minimal, but statistically insignificant, positive correlation was observed between the insertion torque and initial RFA values at the implant placement (r ≠ 0; *p* > 0.05).

## 4. Discussion

The present study aimed to determine whether statistically significant differences are observed between the resonance frequency analysis values measured at the time of implant placement and those measured after the creation of circumferential peri-implant defects in an in vitro model made of a polyurethane block. The primary response variable was the difference between the RFA values measured before and after the creation of the circumferential defect, obtaining an average reduction of almost nine points. Several studies have demonstrated the ability of RFA to measure the primary stability of dental implants [17,33,34,35,36]. Monitoring the primary stability using this system is a method to determine the degree of osseointegration [34,37]. RFA has been largely used for primary stability monitoring to establish implant loading timing, or as an early detection tool in osseointegration failures. However, its use for early detection of peri-implantitis is not yet routinely established. Peri-implantitis is the major biological risk associated with dental implants; however, only a few evaluation methods facilitate the detection of peri-implantitis in the early stages of bone loss. Considering the faster rate of progression of peri-implantitis compared to periodontitis once it is established, it is useful to have early diagnostic instruments to control and limit its spread.

Previous studies have attempted to correlate different types of peri-implant bone defects with the values recorded through RFA. Most of these studies were in vitro studies conducted using different models. Polyurethane blocks with a density of 0.32 gr/cm^3^ were used in the present study owing to their ability to reproduce the mechanical properties of the posterior maxilla. In addition, an external layer of 1 mm thickness with a higher density (1.64 gr/cm^3^) was added to simulate the cortex of the maxillary bone [38,39]. Other types of blocks, such as acrylic resin blocks [40,41], and animal models created using pig [42] or cow ribs [43,44] have also been used to simulate human bone in previous in vitro studies. Human cadaveric models [45,46] and in vivo animal models [34] have also been used in some studies. Notably, only one retrospective study was conducted using a human model [47]. This is because, ethically, only in vitro or animal model studies allow these variables to be analyzed over rational time periods; otherwise, longitudinal cohort studies would have to be carried out, which, although they represent a high level of evidence, are difficult to carry out. Although the bone conditions are best reproduced with real bone, the polyurethane blocks we used have a distinct advantage: they are very homogeneous in terms of shape, density, and elastic properties, which can be very useful when trying to correlate a cause with an effect, as in our case the bone defect with modifications in the RFA, without the presence of covariates or confounding biases.

On the other hand, the sample size of our study (n = 50) is within the range of previously described studies with a similar design and objective to ours, namely between 16 and 84 samples.

The mean initial RFA value of the 50 implants in the present study exceeded 70, with a mean value of 72 in both the BL and MD directions. These results are similar to those obtained in studies with methodologies as varied as in cow ribs [43], Beagle dogs [34], or even in human cadavers [44]. The decrease in RFA values observed in the present study is consistent with the results of other studies that used a similar methodology [40,43]. Similar results were reported for circumferential peri-implant defects by in vitro methodology studies using a human cadaveric model [45]. In this case, a correlation was also found between RFA values and insertion torque. The mean insertion torque value was 29 Ncm, slightly lower than the 32 Ncm in the present study.

A decrease in the ISQ values was also observed in an in vivo animal study that analyzed the variation in implant stability measured by RFA in cases with peri-implantitis [34]. This study, which was conducted on Beagle dogs, revealed that the RFA values decreased when intentionally induced peri-implantitis affected the implants. For this study, thirty-six implants were placed in six Beagle dogs in which peri-implantitis was induced by ligatures, and RFA values were recorded at different times of peri-implant disease development. This finding, which is consistent with the findings of the present study, demonstrates the effect of marginal bone loss on implant stability. However, Sennerby et al. used a prototype RFA device that uses hertz as a unit of measurement, rather than conversion to ISQ values, which was not available at the time of conducting their study [36]. Consequently, the decrease in stability observed in these implants cannot be objectively compared with that observed in the present study.

The RFA values were dependent on the size of the peri-implant defect in previous studies [34,43,46]. However, the degree of correlation between the variables was not consistent across the studies. The greatest drop in RFA values was considered by some experts to occur in the first 2 mm of vertical loss [34,42], whereas more pronounced results were observed from 3 to 4 mm onwards by others. Notably, a practically linear correlation, regardless of the millimeters of loss, was observed in some studies [40,41].

A study analyzing peri-implant dehiscence-type defects revealed that the morphology of the defect is directly correlated with the decrease in RFA values. No correlation between the increase in the depth and the drop in RFA values was observed in cases with narrow dehiscence in this study [44]. These results are consistent with those of another study that reported a statistically significant relationship between the RFA values and peri-implant bone loss in dehiscence-type defects with a depth established at 10 mm, specifically, from a width of 4 mm.

In the present study, the morphology of the peri-implant defect was limited to circumferential defects, 2 mm deep, 8 mm wide, and 2 mm in extension with respect to the neck of the implant. In this sense, many of the articles analyze different depths and widths of the defect. Turkylmaz found a mean RFA value of 68 for defects of the same morphology, slightly higher than that found in the present study [45]. However, this difference could be due to the fact that the sample included anterior and posterior defects, whereas, in our study, only one type of bone feature is simulated. Yao found an RFA value of 60 for narrow circumferential defects of 0.9 mm width and the same depth as ours, slightly lower than the values found in our study [42]. However, the greater length of the implants used—12 mm versus 10 mm in our study—does not allow for realistic comparisons, as the morphology of the implants does not correspond to that used in the current study. Also, in cases of circumferential defects, Medina Madrid has found an average RFA value of 53 [29]. However, in this case, the simulation of the defect is performed in the first third of the implant. Considering that the implants used in this study were 12 mm, this means a defect depth of 4 mm, which is greater than that of our study, and which may justify the higher RFA values found in this study.

Although the differences in reduction between the original group and the defect group are relatively large, with an average of almost 8 ISQ, the final stability of the implants, with an average of 63.38 ISQ, may have little clinical impact. In this context, an experimental in vitro study found that an ISQ of less than 57 could correlate with micromovements of more than 150 µm during loading, which would have a negative impact on the maintenance of osseointegration [47].

Caution should be exercised while using RFA as an isolated diagnostic method for detecting peri-implant marginal bone loss, as the correlation between the two is approximately 1 mm of loss for each unit of decrease in the RFA value, with respect to the basal level of marginal bone [34]. However, this decrease is more pronounced from 4 mm of bone loss [42,45]. For instance, the RFA values decreased by 4 points for each mm of marginal bone loss, and a statistically significant correlation was observed between the two variables from this point onwards. A similar pattern was observed in another study; however, the correlation was not 1:1 as observed in the previous study. The decrease in the RFA values was moderate in circumferential peri-implant defects of up to 3-4 mm, but a decrease of 15 RFA units was observed from this point onwards for each 2 mm of loss. This finding indicates that the difference in implant stability values is most noticeable when peri-implantitis has already been established to depths of >3 mm.

Implants were explanted after measuring implant stability with RFA in a retrospective study that evaluated 37 implants in humans with marginal bone loss deemed to have poor prognoses, that is, those with mobility. The mean RFA value was 37, indicating that explanting implants with peri-implant bone loss and RFA values of <40 is recommended [45]. These results suggest that RFA values can be used to identify implants that will not benefit from receiving conservative treatment. In addition, they can also be used to identify cases of advanced peri-implantitis, wherein the mobility of the implant and its radiographic image indicate a poor prognosis. For this reason, the use of RFA as an early diagnostic tool is still controversial. Routine clinical evaluation of dental implants, including visual examination and periodic probing, can indicate peri-implant status. In the presence of peri-implant inflammation, the role of RFA in determining possible peri-implant bone loss may be an adjunct to periapical radiography.

A correlation between the values of insertion torque and initial RFA has been observed in several studies [41,45,48]; however, this correlation is not statistically significant. This may be attributed to the homogeneity of the protocol used, that is, the use of implants of the same size and identical implantation bed conditions. To compare results between studies, the implant size studied, as well as the morphology and dimensions of the simulated defect, should be standardized.

The RFA values appear to be related to the amount of bone in contact with the implant and its osseointegration [34,37]; however, it should be noted that RFA was designed to measure implant stability [30]. Thus, further extensive studies must be conducted to determine the correlation between the implant stability values and the percentage of bone in contact with the implant. Considering the results obtained in the present study simulating the biomechanical properties of the peri-implant bone environment, its application in an in vivo model and clinical trials could be of interest. However, due to ethical aspects, clinical studies in this regard would only make sense with a retrospective, observational, and descriptive design, which, despite its medium evidence design, could provide relevant conclusions to this proposal. Therefore, histological studies should be reserved only for animal models, which must be incorporated in future study lines, as they can demonstrate the relationship between the decrease in these stability values and the loss of implant osseointegration or peri-implant bone loss.

According to the limitations attributable to the present in vitro study, the absence of measurements in defects of different morphology and size, as well as the use of different implant systems that could resemble the most frequent clinical conditions, are noteworthy.

Even considering the limitations of this in vitro study, the results may lead to recommending the use of RFA in the follow-up of dental implants, firstly because it is a non-invasive test, and secondly, because a decrease in ISQ values during clinical follow-up could indicate the presence of defects in the peri-implant bone support, which could be confirmed by other complementary tests such as probing or radiographs.

## 5. Conclusions

Within the limitations of the present study, it can be concluded that a statistically significant decrease is observed between the values of implant stability measured using RFA at the time of placement and that existing after the simulation of circumferential peri-implant defects in an in vitro model. Further in vivo studies must be conducted in the future to determine the variations in this relationship based on the type and size of the defect, as well as the relationship between the RFA values and the proportion of bone in contact with the implant. In addition, longitudinal studies aimed to correlate decreasing RFA values with radiographic variations to determine a threshold for detecting early marginal bone loss should be conducted.

## Figures and Tables

**Table 1:** Descriptive and inferential statistics of the implant stability results through RFA (ISQ), comparing the two situations analyzed (initial and circumferential defect).

Measurement Direction	Initial vs. Final	Average (ISQ)	Sd	Wilcoxon Signed-Rank Test *p*-Value
BL	Initial	72.23	1.81	*p* < 0.01 *
	Final	63.38	1.76	
MD	Initial	72.25	2.20	*p* < 0.01 *
	Final	63.38	1.49	

* statistically significant.

## Data Availability

The raw data supporting the conclusions of this article will be made available by the authors on request.

## References

[1] Berglundh T., Persson L., Klinge B. (2002). A Systematic Review of the Incidence of Biological and Technical Complications in Implant Dentistry Reported in Prospective Longitudinal Studies of at Least 5 Years. J. Clin. Periodontol..

[2] Albrektsson T., Donos N. (2012). Implant Survival and Complications. The Third EAO Consensus Conference 2012. Clin. Oral Implants Res..

[3] Åstrand P., Ahlqvist J., Gunne J., Nilson H. (2008). Implant Treatment of Patients with Edentulous Jaws: A 20-Year Follow-Up. Clin. Implant Dent. Relat. Res..

[4] Deporter D., Pharoah M., Yeh S., Todescan R., Atenafu E.G. (2014). Performance of Titanium Alloy Sintered Porous-Surfaced (SPS) Implants Supporting Mandibular Overdentures During a 20-Year Prospective Study. Clin. Oral Implants Res..

[5] Moraschini V., Poubel L.A.D.C., Ferreira V.F., Barboza E.D.S.P. (2015). Evaluation of Survival and Success Rates of Dental Implants Reported in Longitudinal Studies with a Follow-Up Period of at Least 10 Years: A Systematic Review. Int. J. Oral Maxillofac. Surg..

[6] Albrektsson T., Zarb G. (1986). The Long-Term Efficacy of Currently Used Dental Implants: A Review and Proposed Criteria of Success. Int. J. Oral Maxillofac. Implants.

[7] Lang N.P., Berglundh T. (2011). On Behalf of Working Group 4 of the Seventh European Workshop on Periodontology. Periimplant Diseases: Where Are We Now?—Consensus of the Seventh European Workshop on Periodontology. J. Clin. Periodontol..

[8] Lindhe J., Meyle J. (2008). On behalf of Group D of the European Workshop on Periodontology. Peri-Implant Diseases: Consensus Report of the Sixth European Workshop on Periodontology. J. Clin. Periodontol..

[9] Chatvaratthana K., Thaworanunta S., Seriwatanachai D., Wongsirichat N. (2017). Correlation between the Thickness of the Crestal and Buccolingual Cortical Bone at Varying Depths and Implant Stability Quotients. PLoS ONE.

[10] Shokri M., Daraeighadikolaei A. (2013). Measurement of Primary and Secondary Stability of Dental Implants by Resonance Frequency Analysis Method in Mandible. Int. J. Dent..

[11] Rakic M., Galindo-Moreno P., Monje A., Radovanovic S., Wang H.-L., Cochran D., Sculean A., Canullo L. (2018). How Frequent Does Peri-Implantitis Occur? A Systematic Review and Meta-Analysis. Clin. Oral Investig..

[12] Atieh M.A., Alsabeeha N.H.M., Faggion C.M., Duncan W.J. (2013). The Frequency of Peri-Implant Diseases: A Systematic Review and Meta-Analysis. J. Periodontol..

[13] Lee C.-T., Huang Y.-W., Zhu L., Weltman R. (2017). Prevalences of Peri-Implantitis and Peri-Implant Mucositis: Systematic Review and Meta-Analysis. J. Dent..

[14] Pimentel S.P., Shiota R., Cirano F.R., Casarin R.C.V., Pecorari V.G.A., Casati M.Z., Haas A.N., Ribeiro F.V. (2018). Occurrence of Peri-Implant Diseases and Risk Indicators at the Patient and Implant Levels: A Multilevel Cross-Sectional Study. J. Periodontol..

[15] Apaza-Bedoya K., Galarraga-Vinueza M.E., Correa B.B., Schwarz F., Bianchini M.A., Magalhães Benfatti C.A. (2024). Prevalence, Risk Indicators, and Clinical Characteristics of Peri-Implant Mucositis and Peri-Implantitis for an Internal Conical Connection Implant System: A Multicenter Cross-Sectional Study. J. Periodontol..

[16] Rodrigo D., Sanz-Sánchez I., Figuero E., Llodrá J.C., Bravo M., Caffesse R.G., Vallcorba N., Guerrero A., Herrera D. (2018). Prevalence and Risk Indicators of Peri-Implant Diseases in Spain. J. Clin. Periodontol..

[17] Monje A., Ortega-Oller I., Galindo-Moreno P., Catena A., Monje F., O’Valle F., Suarez F., Wang H.-L. (2014). Sensitivity of Resonance Frequency Analysis for Detecting Early Implant Failure: A Case-Control Study. Int. J. Oral Maxillofac. Implants.

[18] Hashim D., Cionca N., Combescure C., Mombelli A. (2018). The Diagnosis of Peri-Implantitis: A Systematic Review on the Predictive Value of Bleeding on Probing. Clin. Oral Implants Res..

[19] Ericsson I., Lindhe J. (1993). Probing Depth at Implants and Teeth: An Experimental Study in the Dog. J. Clin. Periodontol..

[20] Lang N.P., Wetzel A.C., Stich H., Caffesse R.G. (1994). Histologic Probe Penetration in Healthy and Inflamed Peri-Implant Tissues. Clin. Oral Implants Res..

[21] Cha J., Wadhwani C., Wang M., Hokett S., Katancik J. (2019). Instrument Selection and Application Used to Probe Dental Implants. Int. J. Oral Maxillofac. Implants.

[22] Schou S., Holmstrup P., Stoltze K., Hjørting-Hansen E., Fiehn N., Skovgaard L.T. (2002). Probing Around Implants and Teeth with Healthy or Inflamed Peri-Implant Mucosa/Gingiva: A Histologic Comparison in Cynomolgus Monkeys (*Macaca fascicularis*). Clin. Oral Implants Res..

[23] Sanz M., Chapple I.L. (2012). On behalf of Working Group 4 of the VIII European Workshop on Periodontology. Clinical Research on Peri-Implant Diseases: Consensus Report of Working Group 4. J. Clin. Periodontol..

[24] García-García M., Mir-Mari J., Benic G.I., Figueiredo R., Valmaseda-Castellón E. (2016). Accuracy of Periapical Radiography in Assessing Bone Level in Implants Affected by Peri-Implantitis: A Cross-sectional Study. J. Clin. Periodontol..

[25] Christiaens V., Pauwels R., Mowafey B., Jacobs R. (2023). Accuracy of Intra-Oral Radiography and Cone Beam Computed Tomography in the Diagnosis of Buccal Bone Loss. J. Imaging.

[26] Gehrke S.A., Marin G.W. (2015). Biomechanical Evaluation of Dental Implants with Three Different Designs: Removal Torque and Resonance Frequency Analysis in Rabbits. Ann. Anat.—Anat. Anz..

[27] Huang H.-M., Lee S.-Y. (2012). Resonance Frequency Assessment of Dental Implant Stability with Various Bone Qualities: A Numerical Approach. Clin. Oral Implants Res..

[28] Koh J.-W., Yang J.-H., Han J.-S., Lee J.-B., Kim S.-H. (2009). Biomechanical Evaluation of Dental Implants with Different Surfaces: Removal Torque and Resonance Frequency Analysis in Rabbits. J. Adv. Prosthodont..

[29] Medina Madrid R., Padullés Roig E., Cabanes Gumbau G., Alarcón Rodríguez R., Boquete Castro A. (2024). ISQ as a Diagnostic Tool in Implants Affected with Bone Loss: An In Vitro Experimental Study. Int. J. Oral Maxillofac. Implants.

[30] Meredith N., Alleyne D., Cawley P. (1996). Quantitative Determination of the Stability of the Implant-Tissue Interface Using Resonance Frequency Analysis. Clin. Oral Implants Res..

[31] Schwarz F., Derks J., Monje A., Wang H. (2018). Peri-implantitis. J. Clin. Periodontol..

[32] Lekholm U., Zarb G.A., Branemark P.I., Zarb G.A., Albrektsson T. (1985). Patient selection and preparation. Tissue Integrated Prsotheses: Osseointegration in Clinical Dentistry.

[33] Monje A., Salvi G.E. (2024). Diagnostic Methods/Parameters to Monitor Peri-Implant Conditions. J. Periodontol..

[34] Monje A., Insua A., Monje F., Muñoz F., Salvi G.E., Buser D., Chappuis V. (2018). Diagnostic Accuracy of the Implant Stability Quotient in Monitoring Progressive Peri-Implant Bone Loss: An Experimental Study in Dogs. Clin. Oral Implants Res..

[35] Meredith N., Books K., Fribergs B., Jemt T., Sennerby L. (1997). Resonance Frequency Measurements of Implant Stability *In Viva*. A Cross-Sectional and Longitudinal Study of Resonance Frequency Measurements on Implants in the Edentulous and Partially Dentate Maxilla. Clin. Oral Implants Res..

[36] Sennerby L., Persson L.G., Berglundh T. (2005). Implant Stability During Initiation and Resolution of Experimental Periimplantitis: An Experimental Study in the Dog. Clin. Implant Dent. Relat. Res..

[37] Scarano A., Degidi M., Iezzi G., Petrone G., Piattelli A. (2006). Correlation Between Implant Stability Quotient and Bone-Implant Contact: A Retrospective Histological and Histomorphometrical Study of Seven Titanium Implants Retrieved from Humans. Clin. Implant Dent. Relat. Res..

[38] Chávarri-Prado D., Brizuela-Velasco A., Diéguez-Pereira M., Pérez-Pevida E., Jiménez-Garrudo A., Viteri-Agustín I., Estrada-Martínez A., Montalbán-Vadillo O. (2020). Influence of Cortical Bone and Implant Design in the Primary Stability of Dental Implants Measured by Two Different Devices of Resonance Frequency Analysis: An In Vitro Study. J. Clin. Exp. Dent..

[39] Ahn S.-J., Leesungbok R., Lee S.-W., Heo Y.-K., Kang K.L. (2012). Differences in Implant Stability Associated with Various Methods of Preparation of the Implant Bed: An In Vitro Study. J. Prosthet. Dent..

[40] Choi H.-H., Chung C.-H., Kim S.-G., Son M.-K. (2014). Reliability of 2 Implant Stability Measuring Methods in Assessment of Various Periimplant Bone Loss: An In Vitro Study with the Periotest and Osstell Mentor. Implant Dent..

[41] Tözüm T.F., Turkyilmaz I., McGlumphy E.A. (2008). Relationship Between Dental Implant Stability Determined by Resonance Frequency Analysis Measurements and Peri-Implant Vertical Defects: An *In Vitro* Study. J. Oral Rehabil..

[42] Yao C., Ma L., Mattheos N. (2017). Can Resonance Frequency Analysis Detect Narrow Marginal Bone Defects Around Dental Implants? An Ex Vivo Animal Pilot Study. Aust. Dent. J..

[43] Shin S.-Y., Shin S.-I., Kye S.-B., Hong J., Paeng J.-Y., Chang S.-W., Yang S.-M. (2015). The Effects of Defect Type and Depth, and Measurement Direction on the Implant Stability Quotient Value. J. Oral Implantol..

[44] Yim H., Lim H.-C., Hong J.-Y., Shin S.-I., Chung J.-H., Herr Y., Shin S.-Y. (2019). Primary Stability of Implants with Peri-Implant Bone Defects of Various Widths: An *In Vitro* Investigation. J. Periodontal Implant Sci..

[45] Turkyilmaz I., Sennerby L., Yilmaz B., Bilecenoglu B., Ozbek E.N. (2009). Influence of Defect Depth on Resonance Frequency Analysis and Insertion Torque Values for Implants Placed in Fresh Extraction Sockets: A Human Cadaver Study. Clin. Implant Dent. Relat. Res..

[46] Chan H.-L., El-Kholy K., Fu J.-H., Galindo-Moreno P., Wang H.-L. (2010). Implant Primary Stability Determined by Resonance Frequency Analysis in Surgically Created Defects: A Pilot Cadaver Study. Implant Dent..

[47] Scarano A., Carinci F., Quaranta A., Iezzi G., Piattelli M., Piattelli A. (2007). Correlation Between Implant Stability Quotient (ISQ) with Clinical and Histological Aspects of Dental Implants Removed for Mobility. Int. J. Immunopathol. Pharmacol..

[48] Brizuela-Velasco A., Álvarez-Arenal Á., Gil-Mur F.J., Herrero-Climent M., Chávarri-Prado D., Chento-Valiente Y., Dieguez-Pereira M. (2015). Relationship Between Insertion Torque and Resonance Frequency Measurements, Performed by Resonance Frequency Analysis, in Micromobility of Dental Implants: An In Vitro Study. Implant Dent..

